# Attitudes and Influential Factors Affecting Medical Students’ Engagement in Research: A Study in the Western Region of Saudi Arabia

**DOI:** 10.7759/cureus.101695

**Published:** 2026-01-16

**Authors:** Inam A Abulreish, Daniyah A Alharbi, Fatimah O Sulaymani, Nusaybah F Alhassani, Abeer Shaker

**Affiliations:** 1 Faculty of Medicine, Umm Al-Qura University, Makkah, SAU; 2 College of Medicine and Surgery, Ibn Sina National College, Makkah, SAU; 3 Pathology Department, College of Medicine, Umm Al-Qura university, Makkah, SAU

**Keywords:** medical students, motivating factors, research barriers, research engagement, undergraduate medical education

## Abstract

Background: Research engagement among medical students is essential for academic and professional development; however, data on students’ attitudes and factors influencing research participation in the western region of Saudi Arabia remain limited. This study aimed to assess attitudes toward research and identify factors influencing research engagement among undergraduate medical students at Umm Al-Qura University and Ibn Sina National College.

Materials and methods: A cross-sectional survey was conducted among second- to sixth-year medical students using a validated, self-administered questionnaire. Data on demographics, research involvement, motivating factors, and perceived barriers were collected. Associations were assessed using chi-square tests, and binary logistic regression was performed to identify predictors of research involvement.

Results: A total of 327 medical students participated; 60.9% were female, and 65.1% were enrolled at Umm Al-Qura University. Although 61.5% reported prior research involvement, only 54.1% were currently engaged in research, while 92.4% expressed willingness to participate in future projects. Admission into residency programs was the most influential motivator (51.4%), followed by competition among students. The most frequently reported barriers were lack of research knowledge (49.2%), time constraints (47.1%), and lack of mentoring (46.5%). Research participation was significantly associated with age (p < 0.001), gender (p = 0.008), and academic year (p < 0.001). Fifth-year students had significantly higher odds of research involvement compared to second-year students (OR = 25.5, p < 0.001). GPA was not a significant predictor in multivariable analysis.

Conclusions: Although most students demonstrated a positive attitude toward research, engagement was primarily associated with career-related factors such as residency program requirements and academic competition. Higher participation among senior students suggests that curricular exposure and academic structure play a key role in facilitating research involvement. Addressing identified barriers through structured mentorship and curriculum-integrated research opportunities may enhance meaningful student participation.

## Introduction

Research is fundamental to medical practice, underpinning evidence-based decision-making and the development of effective diagnostic and therapeutic strategies [1-4]. Early involvement in research during medical training enhances critical thinking, analytical skills, and lifelong learning, while also contributing to professional development and academic competitiveness [5-9]. Consequently, fostering research engagement among undergraduate medical students is widely recognized as an essential component of contemporary medical education.

Moreover, undergraduate research experience has been independently associated with improved academic and career outcomes [9]. Despite the universal benefits mentioned, there exists a significant variation in the extent of undergraduate student involvement in research [10]. Furthermore, it is crucial to foster a positive attitude towards scientific research among students right from the start of their medical career [11]. Research early in their medical training can shape students’ attitudes toward lifelong learning and prepare them for careers in academia or specialized fields [12]. Studies have shown that early exposure to research can significantly enhance students' confidence and competence in conducting studies, which can translate into better patient care and improved healthcare systems [13].

Several studies have identified key factors that strongly motivate medical students to engage in research. These factors include effective mentorship and the opportunity to enhance visibility through the publication of indexed research articles, which are advantageous for future training and career prospects, particularly when aiming to secure a competitive residency spot [1,2]. Conversely, several factors can pose challenges or discourage medical students from engaging in research, including time constraints, an emphasis on education over research, and insufficient training in research methodology [14]. Additionally, a lack of institutional support and limited access to resources can further hinder students' ability to participate in research activities [15].

Previous studies in Saudi Arabia have primarily focused on assessing medical students' knowledge, attitudes, and practices regarding conducting research within single institutions or universities [7,10,11,16]. However, there is a noticeable lack of data regarding medical universities in the western region. This gap in the literature underscores the need for a multicenter study that describes attitudes toward research and associated factors among medical students in this region [17].

Given the observational nature of this cross-sectional study, the findings are intended to describe existing attitudes and associated factors rather than evaluate interventions or policy outcomes. Unlike previous studies conducted at single institutions, this multicenter study focuses on undergraduate medical students in the western region of Saudi Arabia, providing a broader and more generalizable understanding of research attitudes and engagement factors in this context. Accordingly, this study aimed to assess attitudes toward research and identify factors associated with research engagement among undergraduate medical students at Umm Al-Qura University and Ibn Sina National College for Medical Studies. Identifying attitudes toward research and the factors influencing engagement may help inform future educational strategies and guide further research in undergraduate medical education.

## Materials and methods

Study design

This quantitative, cross-sectional study used a convenience sampling method to recruit medical students. Data were collected using a validated self-administered questionnaire, adapted from previously published research [11]. The questionnaire was piloted with 20 students in the western region to ensure clarity, validity, and cultural appropriateness. The study was conducted from November 2023 to March 2025. Participants were invited through social media platforms (X, WhatsApp, Instagram, and Facebook). Participation was voluntary, and completion of all questionnaire items was required for submission. A total of 327 complete responses were obtained and included in the analysis. Measures to reduce bias included anonymous responses, clear instructions, and voluntary participation. Common reasons for non-response included time constraints and lack of interest in research activities. The study focused on medical students from the second to sixth years at Umm Al-Qura University and Ibn Sina National College. Ethical approval for this study was obtained from the Umm Al-Qura University Institutional Research Board (approval number: HAPO-02-K-012-2024-01-1943, approval date: 1 January 2024).

Sample size calculation

The sample size was calculated using the Raosoft® sample size calculator, based on a population of 2,810 medical students from both institutions, a 95% confidence level, and a 5% margin of error. This calculation yielded a required sample size of 339 students. A total of 327 complete responses were obtained, slightly below the target but still sufficient for descriptive and inferential analyses.

Study participants

All second- to sixth-year medical students at Umm Al-Qura University and Ibn Sina National College were included in the study. First-year medical students, medical interns, and students who declined to participate in the study questionnaire were excluded.

Data collection

The data collection process used a structured questionnaire. Questionnaire items were selected from previously validated studies to capture demographic factors, prior research participation, and barriers or motivators to research engagement [11]. This questionnaire was chosen over alternatives because it comprehensively addresses all dimensions of undergraduate research involvement while maintaining prior validation in similar populations. The questionnaire was divided into three sections: the first section collected demographic data, including nationality and education level. The second section assessed research participation, focusing on prior involvement in academic research and willingness to participate. The third section explored barriers and experiences, examining factors that hinder research participation and students’ experiences with research.

Statistical analysis

Statistical analysis was performed using SPSS Statistics version 26 (IBM Corp., Released 2019. IBM SPSS Statistics for Windows, Version 26.0. Armonk, NY: IBM Corp.). Categorical variables were presented as frequencies and percentages. The chi-square test was used to examine associations between research participation (yes/no) and the following sociodemographic variables: gender, year of study, nationality, and GPA. Binary logistic regression was performed to predict the likelihood of research participation (dependent variable) based on statistically significant sociodemographic factors identified in the chi-square analysis (independent variables: gender, year of study, nationality, and GPA). Regression results are presented as odds ratios with 95% confidence intervals and p-values. A p-value <0.05 was considered statistically significant.

Additional information

Regarding the questions, “If you have other barrier(s) that are not listed, please write them down”, “If yes, why is it important?”, and “If not, why is it not important?”, they were open-ended. The most frequent responses were coded, while the less frequent responses were grouped under the category “Other,” which was also coded. For analysis, these questions were included in the descriptive analysis to present frequencies and percentages.

## Results

Sociodemographic data and research involvement

The study surveyed 327 medical students (after excluding non-medical students). The majority were young adult Saudi females. The majority of participants were young adults, female, and Saudi nationals, with a higher representation from Umm Al-Qura University. Research participation increased with academic progression, and most students reported previous research involvement (61.5%) and willingness to participate in future studies (92.4%). Additionally, age, gender, year of study, and GPA were significantly associated with research participation, whereas nationality and university were not (Table 1).

**Table 1:** Association between sociodemographic data and research involvement GPA: grade point average

Parameter	Category	Not involved in research	Involved in research	p-value
		N (%)	N (%)	
Age	18-19	10 (66.7%)	5 (33.3%)	<0.001
	20-21	91 (58.0%)	66 (42.0%)	
	22-23	21 (20.6%)	81 (79.4%)	
	24-25	3 (6.7%)	42 (93.3%)	
	Above 25	1 (12.5%)	7 (87.5%)	
Nationality	Saudi	114 (37.5%)	190 (62.5%)	0.163
	Non-Saudi	12 (52.2%)	11 (47.8%)	
Gender	Male	38 (29.7%)	90 (70.3%)	0.008
	Female	88 (44.2%)	111 (55.8%)	
University	Umm Al-Qura University	78 (36.6%)	135 (63.4%)	0.331
	Ibn Sina National College	48 (42.1%)	66 (57.9%)	
Year of study	Second year	27 (67.5%)	13 (32.5%)	<0.001
	Third year	65 (58.6%)	46 (41.4%)	
	Fourth year	31 (42.5%)	42 (57.5%)	
	Fifth year	3 (4.4%)	65 (95.6%)	
	Sixth year	0 (0.0%)	35 (100.0%)	
GPA category	Weak	4 (57.1%)	3 (42.9%)	<0.001
	Pass	6 (35.3%)	11 (64.7%)	
	Good	11 (22.4%)	38 (77.6%)	
	Very good	18 (23.4%)	59 (76.6%)	
	Excellent	86 (48.9%)	90 (51.1%)	

Motivating factors for conducting undergraduate medical research

The most influential motivators for conducting research among students were admission into residency programs, interest in research as a career, and aiding clinical decision-making (Table 2). Financial return was reported as the least influential factor.

**Table 2:** Motivating factors for conducting undergraduate medical research

Motivating factor	Strongly agree n (%)	Strongly disagree n (%)	Most influential n (%)	Least influential n (%)
Interest in research as a career	117 (35.8%)	20 (6.1%)	-	-
Financial return	-	23 (7.0%)	-	111 (33.9%)
Competition between students	103 (31.5%)	15 (4.6%)	-	-
Admission into residency programs	197 (60.2%)	0 (0.0%)	168 (51.4%)	13 (4.0%)
Aid clinical decision making	143 (43.7%)	5 (1.5%)	-	-
Elective research programs	128 (39.1%)	9 (2.8%)	12 (3.7%)	-

Barriers preventing medical students from participating in undergraduate research

Key barriers included lack of knowledge, time constraints, lack of mentoring, and limited research opportunities. Financial issues and research not relevant to the preferred specialty were less influential (Table 3).

**Table 3:** Barriers preventing medical students from participating in undergraduate research

Barrier	Strongly agree n (%)	Strongly disagree n (%)	Most influential n (%)	Least influential n (%)
Lack of knowledge	161 (49.2%)	3 (0.9%)	83 (25.4%)	25 (7.6%)
Lack of time	154 (47.1%)	4 (1.2%)	54 (16.5%)	43 (13.1%)
Lack of mentoring	152 (47.5%)	4 (1.2%)	37 (11.3%)	14 (4.3%)
Lack of interest in research	124 (37.9%)	10 (3.1%)	-	-
Lack of research opportunities	124 (37.9%)	13 (4.0%)	28 (8.6%)	21 (6.4)
Lack of motivation	122 (37.3%)	8 (2.4%)	-	-
Difficulty obtaining study approval	112 (34.3%)	10 (3.1%)	-	-
Lack of interest in the topic	108 (33.0%)	9 (2.8%)	-	-
Competition over research opportunities	104 (31.8%)	16 (4.9%)	6 (1.8|%)	18 (5.5%)
Limited database access/data	92 (28.1%)	9 (2.8%)	12 (3.7%)	26 (8.0%)
Financial issues	87 (26.2%)	13 (4.0%)	-	-
Research not relevant to the preferred specialty	74 (22.6%)	14 (4.3%)	21 (6.4%)	28 (8.6%)

**Figure 1:**
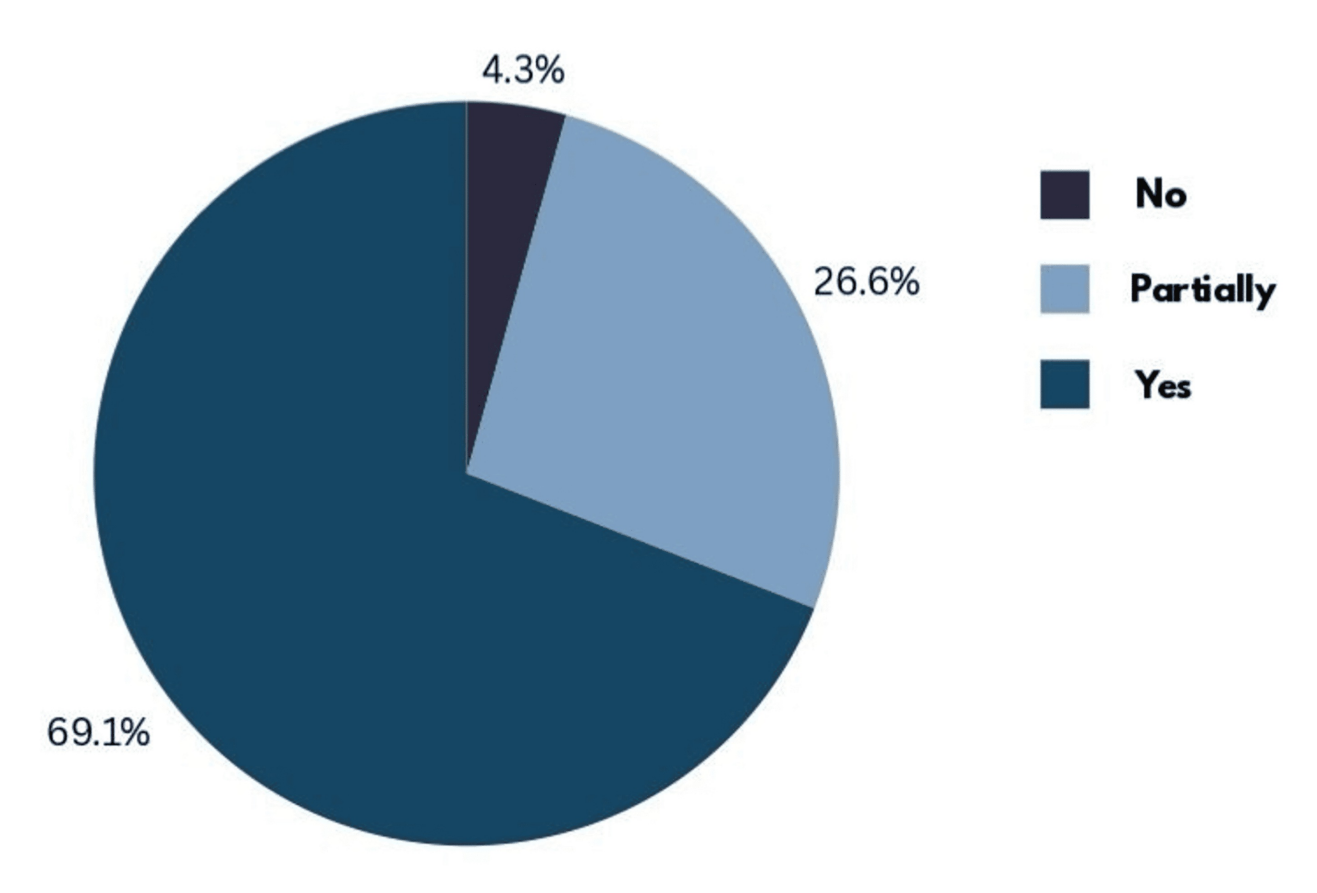
Pie chart illustrating students' perceptions of the importance of research for their careers

The majority (69.1%) considered it important, 26.6% viewed it as somewhat important, and only 4.3% believed it was not important (Figure 1).

**Figure 2:**
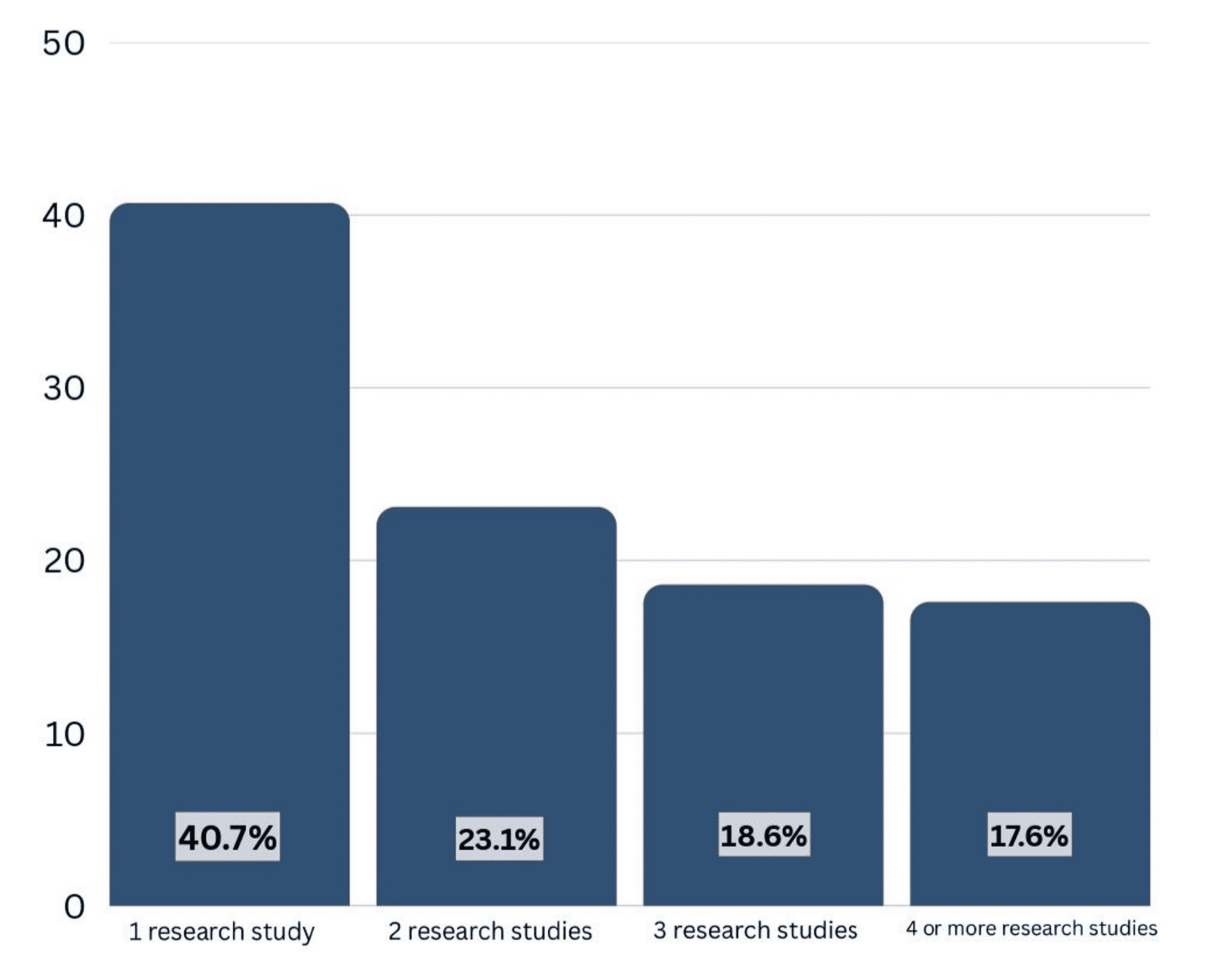
Bar chart illustrating the number of research projects students participated in

The majority of students participated in one to two research projects (approximately 65%), with fewer students engaged in three or more projects (Figure 2).

Predictors of involvement in research based on statistically significant sociodemographic data

Fifth-year students had significantly higher odds of research participation compared to second-year students (OR = 25.55, 95% CI: 5.57-117.21, p < 0.001), while age above 25 showed marginal significance (OR = 11.83, 95% CI: 0.97-143.75, p = 0.052) (Table 4).

**Table 4:** Predictors of involvement in research based on the statistically significant sociodemographic data On a four-point scale, the ranges are the following: weak (2.0–2.4), pass (2.5–2.9), good (3.0–3.4), very good (3.5–3.74), and excellent (3.75–4.0). On a five-point scale, the corresponding ranges are the following: weak (2.5–2.9), pass (3.0–3.4), good (3.5–3.9), very good (4.0–4.4), and excellent (4.5–5.0). GPA: grade point average, OR: odds ratio, CI: confidence interval

Parameter	Category	OR	95% CI		p-value
			LB	UB	
Age	18-19	Ref.	Ref.	Ref.	Ref.
	20-21	0.996	0.257	3.863	0.996
	22-23	2.218	0.508	9.672	0.289
	24-25	2.212	0.235	20.794	0.487
	Above 25	11.832	0.974	143.751	0.052
Gender	Male	Ref.	Ref.	Ref.	Ref.
	Female	0.880	0.480	1.612	0.678
Year of study	Second year	Ref.	Ref.	Ref.	Ref.
	Third year	1.737	0.686	4.398	0.244
	Fourth year	2.121	0.765	5.880	0.149
	Fifth year	25.547	5.569	117.205	<0.001
GPA category	Weak	Ref.	Ref.	Ref.	Ref.
	Pass	2.087	0.202	21.576	0.537
	Good	3.996	0.495	32.260	0.194
	Very good	5.219	0.697	39.088	0.108
	Excellent	1.937	0.272	13.801	0.509

## Discussion

Our research aims to evaluate and understand the factors that influence medical students’ participation in scientific research. A total of 327 participants were included in the study, comprising 213 students from Umm Al-Qura University and 114 students from Ibn Sina National College. The data revealed that medical students were primarily motivated to pursue research by their desire to enter residency programs in Saudi Arabia, competition with peers, and the opportunity to enhance their clinical decision-making skills. However, common barriers they encountered included insufficient knowledge of research methods, limited time availability, and a lack of supervision.

While residency admission drives participation (60.2%), this instrumental motivation may undermine long-term research engagement. Programs should therefore foster intrinsic interest through early exposure, such as first-year research electives. This study explored factors influencing medical students’ participation in research, and our findings suggest that engagement in research is affected by academic year, age, and GPA. Senior students (fifth- and sixth-year) were considerably more involved than junior students. These results align with previous studies, indicating that senior students are more likely to participate in research due to greater exposure and the recognition of its importance for career progression [6,7].

For instance, a study by Awofeso et al. (2020) in Nigeria found that senior medical students were more engaged in research activities because of the perceived advantages for career advancement, particularly when applying for residency programs [6]. Similarly, Alhabib et al. (2023) reported that final-year medical students exhibited markedly increased levels of research involvement, driven by their recognition of research as a crucial factor in securing competitive residency placements [7]. This closely aligns with our findings, in which 60.2% of students strongly agreed that research participation was essential for admission to residency programs.

Furthermore, a systematic review and meta-analysis by Amgad et al. (2015) highlighted that medical students who participated in research were more likely to view it as beneficial for career progression, particularly concerning residency applications [18]. This reinforces the significant influence of residency admission requirements on students' motivation to engage in research.

Among the factors influencing research engagement, 35.8% of participants strongly agreed that they had a strong interest in research as a career. Previous research has demonstrated that students who expressed such interest were more likely to participate in research activities during their training, underscoring the importance of cultivating this interest early [19]. Another significant motivator was competition among peers, with 31.5% strongly agreeing. This suggests that competitive environments may encourage students to conduct research to strengthen their résumés and improve their prospects for residency placement [20].

Admission into residency programs was identified as the most influential motivator, with 51.4% selecting it as the most important factor overall. Participation in research enables medical students to attend scientific conferences and competitions, thereby increasing their involvement within the academic medical community. This engagement also enhances their chances of acceptance into residency or training programs, as it contributes to the curriculum vitae points required by the Saudi Commission for Health Specialties, as outlined in the 2024 matching system booklet. A study conducted at Alfaisal University reported that 91% of students were motivated to participate in research to meet residency program requirements, emphasizing the pivotal role of research in improving residency applications [21].

The barriers to research engagement identified in our study included a significant lack of genuine research interest. One study indicated that many medical students engage in research primarily to enhance their residency applications rather than from intrinsic motivation [22]. Additionally, barriers such as lack of knowledge, limited time, and insufficient mentorship were consistent with findings from other studies. Ommering et al. (2018) reported that medical students encountered barriers such as restricted access to research opportunities and inadequate mentoring [2]. Similarly, Bonilla-Escobar et al. (2017) highlighted insufficient research guidance and the challenge of balancing clinical work with research responsibilities as major obstacles [1]. These findings emphasize the need for medical colleges to provide structured mentorship and integrate research opportunities within the medical curriculum to overcome these limitations.

Another notable finding was the critical role of mentorship in research participation. A substantial proportion of students (46.5%) strongly agreed that a lack of mentoring represented a significant barrier. This aligns with previous studies emphasizing the crucial role of mentorship in fostering research participation. For example, a study of doctors in India found that 37.39% experienced difficulties due to a lack of proper guidance or mentorship. Similarly, research on medical students identified a lack of mentoring and guidance as the main barrier to conducting research [23,24].

Both lack of motivation (37.3%) and time restrictions (47.1%) also discouraged research participation. Limited time may lead students to prioritize academic studies over research activities, while a lack of motivation may hinder their ability to engage effectively in research writing. This aligns with findings by Al-Subai et al. (2014), who observed that medical students prioritize their coursework over research activities [25]. A comprehensive review further noted that time constraints and lack of motivation are common barriers to research participation among medical students, suggesting that these issues are widespread [26].

In contrast, Rosenkranz et al. (2015) found that time constraints were less limiting when structured research electives were embedded within the medical curriculum, allowing students to dedicate time to research activities [5]. Another barrier identified was the difficulty in obtaining study approval, with 34.3% of respondents strongly agreeing that this factor restricted their research participation.

Moreover, the lack of financial support was cited as a barrier by 26.6% of participants who strongly agreed and 29.4% who agreed, including students from Umm Al-Qura University and Ibn Sina National College for Medical Studies. This finding aligns with that of Alhabib et al. (2023), who found that financial constraints significantly limited students' participation, particularly when students were required to self-fund research projects or cover costs for conferences and publications [7]. Similarly, Sobczuk et al. (2022) emphasized that financial support is critical to enabling students to participate in research, especially in settings where such costs are not subsidized [14]. Overall, these findings confirm that financial barriers can substantially deter students from pursuing research opportunities [27].

Choosing a research topic also emerged as a significant barrier, with 71.4% of students reporting it as a challenge. Similar findings were reported by Mayne et al. (2024), who found that medical students felt overwhelmed by the lack of guidance in choosing a topic relevant to their field [28]. This suggests that guiding topic selection and aligning research projects with students' interests and career goals could enhance research involvement.

Consistent with our findings, lack of knowledge is a significant barrier to research participation. One study reported 90.7% of students cited this obstacle [26]. Recent evidence from Pakistan reported similar challenges among undergraduate medical students [29]. Differences may reflect variations in study populations, curricula, prior research experience, or survey design. Nonetheless, all studies indicate that insufficient knowledge remains a key impediment to student engagement in research.

Demographic factors were also found to influence research participation. Age was a significant determinant, with older students more likely to engage in research. This finding aligns with evidence that maturity and academic experience enhance motivation and performance in research activities [30].

Consistent with previous studies [7,30]. Our results showed that senior students were more likely to participate in research. This may be due to increased exposure to research opportunities, curricular requirements, and recognition of its importance for residency applications. Similarly, students with higher GPAs were more engaged in research, suggesting that academic excellence may be associated with better time management, motivation, and access to research resources. These findings align with prior evidence indicating that high-achieving students tend to participate more in extracurricular activities, including research. The introduction and discussion have been updated to consistently reflect the positive association between GPA and research involvement in our study.

Finally, 92.4% of participants expressed willingness to participate in future research, reflecting a highly positive attitude among medical students towards research. This suggests substantial potential for increased engagement if existing barriers are effectively addressed. Similar findings were reported by Bonilla-Escobar et al. (2017), who found that medical students demonstrated a strong research interest. However, their study also emphasized that, despite this willingness, barriers such as limited research opportunities and inadequate mentorship often prevented students from translating interest into active participation [1]. Addressing these barriers through structured programs and improved access to resources could effectively leverage this enthusiasm, thereby enhancing research productivity.

Limitations

One limitation of this study was that it assessed medical students from two universities in the western region of Saudi Arabia, which may restrict the extent to which the findings can be applied beyond this specific geographical area. Future studies should incorporate participants from multiple universities across different regions of Saudi Arabia and use more accurate measures than questionnaires to collect data.

Implications and recommendations

Our research suggests that medical schools should prioritize engaging their students in research opportunities and implementing monitoring programs to track students’ progress and provide guidance. One recommendation is to integrate curriculum changes by dedicating four to six weeks annually for research. Additionally, establishing mentorship networks to pair students with faculty via institutional platforms can enhance support. Finally, schools should consider subsidizing conference travel and publication fees for student-led projects. These steps will foster an environment of active participation and academic excellence in medical research.

## Conclusions

Our study showed that while the majority of students exhibited a positive attitude toward research, the main factors influencing this attitude were acceptance into residency programs and competition among students, rather than a genuine interest in the research field. The main barriers encountered were a lack of knowledge, insufficient time, and insufficient mentoring. Medical schools need to enhance their research methodology curriculum by devoting more time to research and adopting engaging teaching methods. Moreover, having mentors to guide and inspire students would increase participation in research. By addressing these barriers and creating a positive environment, schools can motivate students to engage in and excel at research.

## Appendices

Questionnaire

Are you a medical student? (If they answer no, it ends the survey)

○           Yes

○           No

(If participants answer no, the survey will take them to a section saying: “Thank you for your time. Unfortunately, our research is limited only to medical students. Please click on 'submit' before exiting.” If participants answer yes, they will continue with the survey questions.)

What is your age? Drop-down menu

What is your nationality?

○           Saudi

○           Non-Saudi

Gender

○           Male

○           Female

What university do you study at?

○           Umm Al-Qura University

○           Ibn Sina National College

○           Other: please specify

What year are you currently in?

○            Second year

○            Third year

○           Fourth year

○           Fifth year

○           Sixth year

What is your current GPA? (out of 4)

○           2.0-2.4

○           2.5-2.9

○           3.0-3.4

○           3.5-3.74

○           3.75-4.0

○           It's out of 5

If it's out of 5, please choose one of the following.

○           2.5-2.9

○           3.0-3.4

○           3.4-3.9

○           4.0-4.4

○           4.5-5.0

Have you been involved in research before?

○           Yes

○           No

Are you currently working on a research project?

○           Yes

○           No

Are you willing to participate in a future research project?

○           Yes

○           No

Please select the degree to which the stated factors play a role/motivate you to conduct undergraduate medical research:

Lack of interest in research

○           Strongly agree

○           Agree

○           Neutral

○           Disagree

○           Strongly disagree

Research is not relevant to my preferred specialty

○           Strongly agree

○           Agree

○           Neutral

○           Disagree

○           Strongly disagree

Lack of knowledge

○           Strongly agree

○           Agree

○           Neutral

○           Disagree

○           Strongly disagree

Lack of research opportunities

○           Strongly agree

○           Agree

○           Neutral

○           Disagree

○           Strongly disagree

Lack of time

○           Strongly agree

○           Agree

○           Neutral

○           Disagree

○           Strongly disagree

Lack of mentoring

○           Strongly agree

○           Agree

○           Neutral

○           Disagree

○           Strongly disagree

Financial issues

○           Strongly agree

○           Agree

○           Neutral

○           Disagree

○           Strongly disagree

Limited database access/data

○           Strongly agree

○           Agree

○           Neutral

○           Disagree

 ○          Strongly disagree

Lack of interest in the topic

○           Strongly agree

○           Agree

○           Neutral

○           Disagree

○           Strongly disagree

Lack of motivation

○           Strongly agree

○           Agree

○           Neutral

○           Disagree

○           Strongly disagree

Competition over research opportunities

○           Strongly agree

○           Agree

○           Neutral

○           Disagree

○           Strongly disagree

Difficulty obtaining approval for the study

○           Strongly agree

○           Agree

○           Neutral

○           Disagree

○           Strongly disagree

Of the factors stated previously, which would you say has the MOST influence?

○           Drop-down menu with the same factors as the question

Of the factors you picked in the previous question, which would you say has the LEAST influence?

○           Drop-down menu with the same factors as the question

If you have other barrier/s that are not listed, please write them down.

○            Must be limited to 15 words

Are these motivators and barriers based on your previous research experience?

●         Yes or No

●         If yes, how many research studies have you participated in?

○         1 research study

○         2 research studies

○         3 research studies

○         4 or more research studies

●         How many articles have you published?

○         None

○         1 publication

○         2 publications

○         3 publications

○         4 or more publications 

Is research important for your career?

○           Yes

○           Partially

○           No

If yes, why is it important?

If not, why is it not important?
